# Impact of orthoses on imagined and actual walking

**DOI:** 10.1186/1757-1146-7-S1-A69

**Published:** 2014-04-08

**Authors:** C Puil, M Janin

**Affiliations:** 1 Applied Podiatry College, 7 Treguel, 86000 Poitiers, France; 2 Podiatrist, Office, 64 Emile Zola, 44550 Saint Malo de Guersac, France; 3 Podiatrist, PhD, Clinic, 7 Treguel, 86000 Poitiers, France

## Background

We know that duration between an actual movement and an imaginary one is similar [1]. We wondered if wearing insoles has an impact on those timings, and particularly if different kinds of insoles have a different impact. We do know that walking changes the respond of motor commands [2]. Feet have a real role in walking as we know. Wearing insoles have an effect on the biomechanics of the entire body [3]. Two kinds of corrections exist. Biomechanical insoles correct with higher stimulations than mediation postural ones. The remediation uses different ways of informations regarding the insoles and so is the effective response.

To compare we have made for each patient a pair of mediation postural foot orthoses (FosMP) and a pair of biomechanical foot orthoses (FosBM). 10 patients have walked actual 30 feet on physical practice (PP) and imagined walking, mental practice (MP).

We have tested them in tree conditions: without insoles (control Ct), with FosMP and with FosBM. We have looked the FosMP/BM modifications through platform’s parameters: speed, speed’s variation and area of center of force; bio-clinical evaluation: posturodynamic test [4] and visual-analogic-scale to evaluated their comfort. All parameters were recorded before (Pre) and after (Post) PP and MP.

Results expose duration of walking in MP and PP.

## Results

**Figure 1:**
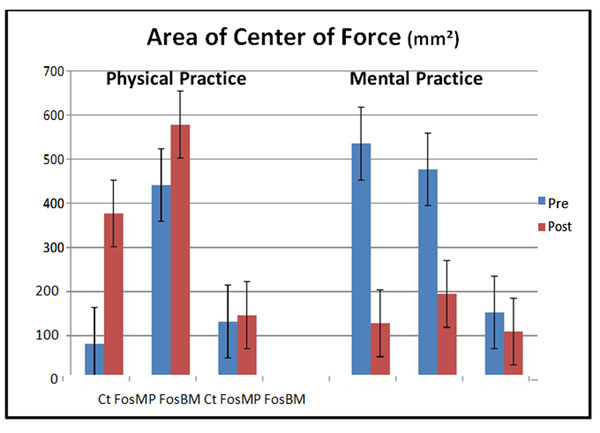


We can see through the results that speed is less important with FosBM (9,917mm.s^–1^ σ=5,757) than with FosMP (12,851 mm.s^.1^ σ=11,013) on post walking in all conditions PP and MP (figure 1).

Post PP walking shows higher results on speed than pre ones (22,068mm/s, σ=14,973; 12,851mm/s, σ=11,013).

Post MP walking are the opposite (8,283mm/s, σ=6,690; 10,397mm/s, σ=8,095)

**Figure 2:**
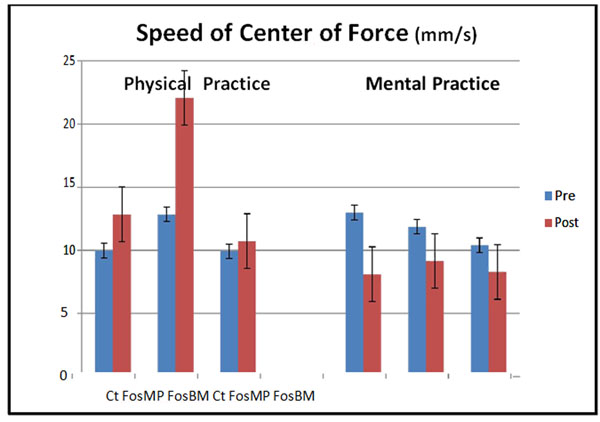


Area of center of force is increased after PP (441,571mm², σ=826,595; to 578,466mm², σ=509.770) (figure 2). MP decreases these area (476,951mm², σ=807,630; 194,768mm², σ=333.557). Speed variation of center of force also shows that PP walking makes post measures increase significantly (68,708mm/s, σ=48,781; 118.02mm/s, σ=45,187).

## Conclusions

We can conclude that wearing FOsBM stabilized patients. Indeed after walking they have a smaller surface of oscillations. They also have a less important speed: motor imagery control is dominant. Wearing FOsBM seems to use less energy in movement or recuperation.

PP makes speed and speed variation of center of force increase, on the contrary of MP.

Those new informations can be useful in sport or reeducation. We know that maintaining body segments increase the body capacity by slowing down his movements, especially after an effort.
